# Lockdown Fatigue in Pediatric Respiratory Patients: Lessons from the First COVID-19 Year

**DOI:** 10.3390/children9121862

**Published:** 2022-11-30

**Authors:** Michal Cahal, Israel Amirav, Mika Rochman, Moria Be’er, Omri Besor, Moran Lavie

**Affiliations:** Pediatric Pulmonology Unit, Dana-Dwek Children’s Hospital, Tel-Aviv Sourasky Medical Center, Sackler Faculty of Medicine, Tel Aviv University, 6 Weizman Street, Tel Aviv 6423906, Israel

**Keywords:** COVID-19, lockdown fatigue, chronic respiratory disorders, children, anxiety, sedentary lifestyle

## Abstract

Lockdown policies have been implemented worldwide to limit the spread of COVID-19. “Lockdown fatigue” is a state of exhaustion related to the accumulating effects of repeated lockdowns. The aim of the current study was to examine the effects of repeated lockdowns on children with respiratory disorders. Data on children aged 0–18 years old with respiratory disorders were collected by an electronic survey during the third lockdown in Israel. The retrieved information included demographics and baseline medical status, respiratory clinical status during the third lockdown compared to pre-lockdown periods, lockdown adherence, lifestyle modifications and caregiver emotional status. The results were compared to those of a similar questionnaire distributed during the first lockdown. A total of 234 patients (62% males, 37% females, mean age 6.8 years (confidence interval 6.1–7.5)) were enrolled. Respiratory symptoms and exacerbation frequency were reduced in 76 (35.5%) and 58 (27.4%) patients, respectively, compared to the pre-lockdown period and similar to the first lockdown (*p* = 0.840 and *p* = 0.063, respectively). However, compared to the first lockdown, the third lockdown was associated with greater use of reliever medications (*p* = 0.006), less use of inhaled corticosteroids as routine treatment (*p* = 0.027), and more pediatric emergency room visits and hospitalizations (*p* = 0.001 and *p* < 0.001, respectively). The results also showed an increase in sedentary lifestyle (e.g., reduced physical activity (*p* = 0.025), less healthy eating habits (*p* = 0.001)) and reduced adherence to lockdown policies (*p* < 0.001). These data show that the continuing stability of clinical improvement during lockdown was accompanied by deleterious sequelae that potentially indicate “lockdown fatigue” among children with respiratory disorders.

## 1. Introduction

COVID-19 was first detected in Wuhan city, China in late December 2019 [1] and soon resulted in a worldwide pandemic [2]. Lockdowns are among the drastic measures that have been implemented to limit the spread of the virus [3], whose beneficial effects [4] also bear worrying implications [5]. Lockdowns have been shown to impact multiple aspects in the pediatric population, such as attention deficit disorder [6], lifestyle alterations, obesity [7], endocrinological aspects [8] and healthcare utilization [9]. Data on the effects of lockdowns on children with respiratory disorders are emerging as well. For instance, pediatric asthma patients exhibited a decline in hospital asthma admission rates during lockdown periods [10]. It has been suggested that this decline may be related to the reduction in the incidence of viral respiratory tract infections [11], improvement in adherence to treatment [12] as well as global reduction in air pollution [10,13].

Our previous work, undertaken during the first COVID-19 national lockdown, focused on the short-term effects of lockdown policies. Our results demonstrated clinical improvement in children with respiratory disorders during the first COVID-19 lockdown, particularly among those younger than 5 years of age and among asthmatic patients. In contrast, patients older than 5 years of age were more likely to experience clinical worsening during the lockdown along with a significant increase in sedentary lifestyle seen in this subgroup. Caregiver anxiety levels were also observed to be elevated [14] and they were included among the emotional implications of lockdowns [15,16].

Repeated lockdowns are taking place worldwide, increasing the risks for accumulating effects on the population and resulting in “lockdown fatigue” [17] defined as a state of exhaustion related to the long-term effects of daily living with COVID-19 lifestyle modifications [18]. Previously published works noted effects of “lockdown fatigue” related to the impact of social distancing in increments on depression and anxiety disorders [19,20], cognitive impairment [21], as well as generalized fatigue [22]. In search for solutions, the government of Australia and even the World Health Organization already published advised measures in order to cope and receive professional help for “lockdown fatigue” and mental health [18,23].

Despite the cautious optimism about the initiation of worldwide vaccinations against COVID-19 [24] and the promising results already observed in Israel [25], the COVID-19 pandemic and, accordingly, repeated lockdowns will apparently constitute the reality in the foreseeable future, especially since new COVID-19 mutations continue to emerge [26,27].

We hypothesized that repeated lockdowns and lifestyle modifications due to COVID-19 may have accumulating effects on children with respiratory conditions. The aim of the current study was to examine the effects of accumulating lockdowns on pediatric respiratory patients over the first year of the COVID-19 pandemic by comparing the findings on the same selected parameters on a questionnaire distributed during the first lockdown with those on the same questionnaire filled in during the third one.

## 2. Methods

### 2.1. Subjects

Eligibility criteria: Children aged 0–18 years old (here after referred to as patients) with a respiratory condition who were followed at the Pediatric Pulmonology Unit Dana-Dwek Children’s Hospital, Tel Aviv Medical Center, Israel were eligible for study entry. Those for whom the complete data on both electronic questionnaires were available were recruited (Supplementary File S1).

This study was approved by the local ethics committee (TLV-0222-20). Informed consent was obtained by the caregivers’ agreement to enter the website and their willingness to complete the online questionnaire.

### 2.2. Data Collection

There were three lockdown periods during the first year of COVID-19 in Israel: the first took place from 15 March to 17 May 2020 (9 weeks), the second from 17 September to 18 October 2020 (4 weeks) and the third from 8 January to 11 February 2021 (4 weeks). Anonymous electronic questionnaires were distributed directly to the caregivers of all of our patients via short message services, one during the first lockdown and the other during the third one. The questionnaires of the first and third lockdowns were identical except for one additional question related to infection (contagion) with COVID-19 in the latter. The caregivers were asked to compare clinical status and lockdown parameters of the patients to non-lockdown (ordinary) periods. Data collected from the questionnaires were distributed into four main sections. The first queried about the child’s demographic and baseline medical status (e.g., age, sex, respiratory disorder, regular respiratory treatments, comorbidities, etc.). The second covered the child’s clinical status during the lockdown compared to non-lockdown status (improved, unchanged or worsened). This was assessed by three key questions: (a) respiratory symptoms, (b) frequency of exacerbation of respiratory symptoms, and (c) use of reliever medications. Additionally queried were changes in adherence to routine respiratory treatment and infection with COVID-19. The third dealt with lockdown-related parameters such as adherence to current lockdown policies, lifestyle modifications and utilization of medical services, and the fourth covered the caregivers’ emotional status during the lockdown (a detailed description of the questionnaire items is provided in our previous work [17]).

The primary outcome was the patient’s clinical status during the third lockdown compared to the first lockdown. The secondary outcomes were changes in the patient’s lifestyle and in the caregiver’s emotional status between the first and third lockdowns.

### 2.3. Statistical Analyses

The patients’ demographics were assessed with descriptive statistics. Pearson’s chi-squared test or Fisher’s exact test was performed to assess correlations among changes in clinical status during lockdown, lockdown variables and caregiver perspectives. Similar tests were later performed to assess correlation between these variables in the first and in the third lockdown. A subgroup analysis was performed only for the patients diagnosed with asthma. Continuous variables are displayed as means (standard deviation [SD]), and their results were compared by a repeated measures t-test. The statistical analysis was performed with IBM SPSS^®^ statistics 25 (Chicago, IL, USA).

## 3. Results

A total of 234 caregivers completed the questionnaire during the third lockdown.

### 3.1. Demographic and Baseline Medical Status

The demographic characteristics of the patients suggested a representative sample of our pulmonary clinic patients. These characteristics were similar for the first and third lockdowns (Table 1) [14]. The sex distribution of 62% males and 38% females (*p* = 0.838) was also similar. The mean age of the patients during the first and third lockdowns was 6.3 (CI 5.4–6.9) and 6.8 (CI 6.1–7.5) years, respectively. The baseline medical status was also similar, with the only differences being a higher prevalence of recurrent pneumonia as the respiratory disorder in the first lockdown compared to the third lockdown (21.5% vs. 15%, respectively, *p* = 0.039) and a higher prevalence of patients with bronchopulmonary dysplasia in the third lockdown compared to the first (12% vs. 7.2%, respectively, *p* = 0.036). There was greater use of inhaled corticosteroids (ICS) as routine treatment during the first lockdown compared to the third lockdown (48.7 vs. 39.7%, respectively, *p* = 0.027). In addition, asthma and allergic disorders were more prevalent among children older than 5 years of age during the third lockdown (62.6% vs. 37.4%, *p* = 0.005 and 87.5% vs. 12.5%, respectively, *p* < 0.001).

### 3.2. Clinical Status during Lockdowns

Clinical status was defined by respiratory symptoms, exacerbation frequency and use of reliever medications. Respiratory symptoms and exacerbation frequency were reported as having improved during both the first and third lockdowns (respiratory symptoms (33% and 27.4%, respectively), *p* = 0.063; exacerbation frequency (35.5% in both lockdowns), *p* = 0.844). In contrast, the caregivers reported increased use of reliever medications during the third lockdown compared to the first (12.2% vs. 21.7%, *p* = 0.006) (Table 2).

We had identified cofactors which were significantly associated with clinical improvement in all three key questions (respiratory symptoms, frequency of exacerbation of respiratory symptoms and use of reliever medications) during the first lockdown [17]. Those cofactors included age of <5 years and a diagnosis of asthma, allergy and multiple respiratory disorders, and they were absent during the third lockdown. However, there was an increased frequency of symptom exacerbation in patients older than 5 years of age compared to those younger than 5 years of age during the third lockdown (*p* < 0.001 and *p* = 0.005, respectively). Seven patients (2.9%) were reported to have positive evidence of infection with COVID-19 during the pandemic, and six of them (85.7%) had asthma as their underlying respiratory disease.

### 3.3. Lockdown-Related Parameters

Adherence to lockdown policies was significantly lower during the third lockdown compared to the first lockdown. During the first lockdown, 331 (74.2%) patients fully adhered and 109 (24.4%) adhered satisfactorily, whereas only 136 (58.6%) fully adhered and 86 (37.1%) adhered satisfactorily (*p* < 0.001) during the third lockdown.

Utilization of medical services was similar for the two lockdowns; however, there was a shift to remote medicine manifested by increase in the usage of telephone/video chats and e-mail services during the third lockdown compared to the first one (26.5% vs. 39.7%, *p* < 0.001 and 7% vs. 29.9%, *p* < 0.001, respectively), as well as increased emergency room (ER) visits and hospitalizations during the third lockdown compared to the first (2.9% vs. 8.5%, *p* = 0.001 and 0.9% vs. 6%, *p* < 0.001, respectively).

A sedentary lifestyle was prevalent in both lockdowns. While 20% of the responders reported increased physical activity in the first one, the figure dropped to 12% in the third one (*p* = 0.025), and there was a similar trend in lower consumption of a healthier diet (32.6% vs. 19.4%, *p* = 0.001) (Table 3). Specifically, patients older than 5 years of age underwent a significant increase in sedentary lifestyle during the third lockdown compared to pre-lockdown periods, including increased screen time, decreased physical activity and shorter sleep duration (*p* < 0.001, *p* < 0.001, and *p* < 0.005, respectively). These data are similar to those reported by us during the first lockdown [17].

### 3.4. Caregivers’ Emotional Status

Elevated caregiver anxiety and risk perception levels were similar for both lockdowns, with over 50% of caregivers reporting increased anxiety compared to the pre-lockdown period (58% vs. 53.5%, *p* = 0.056), and roughly 70% reporting perceptions of increased risk for their child (72.8% vs. 68.8%, *p* = 0.456) during the first lockdown compared to the third.

There were no statistical correlations between any of the various study measures (e.g., demographics, clinical status or lockdown-related parameters).

## 4. Discussion

This study focused on the effects of multiple lockdowns on pediatric respiratory patients throughout the first year of the COVID-19 pandemic. Our results showed that the overall improvement in their clinical status during the lockdowns compared to pre-lockdown periods that we had observed earlier [17] was mostly sustained. However, they also revealed decreased use of routine treatment and increased use of reliever medications during the third lockdown compared to the first one, increased pediatric ER visits and hospitalizations, augmentation of a sedentary lifestyle as well as reduced adherence to lockdown policies.

The clinical improvement in our patients was manifested by reduced respiratory symptoms and frequency of exacerbations during the third lockdown compared to the pre-lockdown period. This improvement is in concordance with our previous results from the first lockdown and may be explained by our speculation on decreased exposure to pollens, pathogens and air pollution due to home confinement during lockdowns, which is probably relevant to multiple lockdowns to a similar extent [14]. However, we did identify a partial worsening in clinical status during the third lockdown, manifested by increased use of reliever medications as well as increased pediatric ER visits and hospitalizations compared to our data from the first lockdown. This may be explained by the reduced adherence to lockdown policies, reduction in the usage of routine ICS, further increments in sedentary lifestyle and timing of lockdown (i.e., during the winter) as seen in our data from the third lockdown. These factors may have hampered the beneficial effect of the decreased environmental exposures and might be related to the occurrence of “lockdown fatigue” [18].

Reduced adherence to lockdown policies can be clarified by the protection motivation theory (PMT), which attempts to explain appraises to potential threats and includes four main components: threat severity, probability of occurrence, self-efficacy perception and efficacy of recommended protective behavior [28]. Evaluation of PMT strategies during the COVID-19 pandemic by Okuhara et al. revealed that threat severity and perception of self-efficacy were stronger predictors of consenting to “staying at home policies” [29], hence a relatively “mild” threat severity in children from infection with COVID-19 could provide an explanation to the reduced adherence to lockdown policies in our patients.

Reduced adherence to lockdown policies might have resulted in decreased home confinement followed by increased exposure of children to one another as well as to adults and therefore to respiratory pathogens, as well as to pollen and air pollution acting as triggers for respiratory exacerbations [30,31].

Reduced perception of threat severity may also explain the reduction in the use of routine treatment of ICS, an important factor in asthma control [32]. Low adherence to routine care during the third lockdown may provide an additional explanation for the increased use of reliever medications, ER visits and hospitalization rates.

Another significant factor was the increment in sedentary lifestyle. Caregivers reported further augmentation of sedentary habits during the third lockdown compared to the first one, which could be correlated to the well-known association of poor asthma control and sedentary lifestyle [33,34]. This is especially relevant to patients older than 5 years of age, who were more likely to worsen clinically during the third lockdown and in line with global results of the worrisome effects of increased sedentary behaviors during COVID-19 lockdowns [35,36], supporting the propensity for clinical worsening and increased sedentary lifestyle observed by us earlier [14].

The season in which the third lockdown took place may also have influenced our current findings. The third lockdown was implemented during the mid-winter months in Israel (January to February). Despite the reduction in respiratory morbidity during lockdowns [37,38], it is possible that the combination of reduced adherence to lockdown policies and therefore increased exposure of children to others could serve as a source for resurgence of respiratory viral infections that more commonly occur during the winter, leading to respiratory exacerbations [39].

Interestingly, while the utilization of medical services during the third lockdown remained relatively stable compared to the first lockdown, we noticed a significant increment in the use of remote medicine. Our results correlated with emerging data on the use of remote medicine during the COVID-19 pandemic and its advantages in patient surveillance and protection [40].

The strength of this study includes a large number of participants and the ability to use similar outcome measures between lockdowns. To the best of our knowledge, it is the only study which addressed the concern of lockdowns fatigue and helped to fill the knowledge gap in this area. We are aware of several limitations: the study lacks objective measures to assess clinical status since the data were obtained during lockdowns and we were not able to perform relevant tests such as pulmonary function tests.

In conclusion, the clinical status in children with respiratory disorders remained stable or improved during multiple lockdowns for the COVID-19 pandemic. At the same time, there was a significant increase in the use of reliever medications, ER visits and hospitalizations during the third lockdown. These findings may be explained by reduced adherence to lockdown policies, reduced use of routine treatments and further increase in a sedentary lifestyle suggesting a potential effect of “lockdown fatigue”. Despite the initial worldwide necessity for social distancing and lockdowns [41], it appears that the effectiveness and long-term consequences of such measures are questionable.

“Take home message”: Clinical status of children with respiratory disorders improved during COVID-19 lockdowns compared to pre-lockdown periods. However, it was accompanied by deleterious clinical and lifestyle sequelae that potentially indicate “lockdown fatigue”.

## Figures and Tables

**Table 1:** Demographic and baseline medical status.

		Lockdown				
		First		Third		
		Patients (*n*)	Patients (%)	Patients (*n*)	Patients (%)	*p* Value
Sample size		445		234		
Sex	Male	276	62.0	147	62.8	0.838
	Female	169	38.0	87	37.2	
Respiratory disorder	Asthma	291	65.2	140	59.8	0.164
	Recurrent pneumonia	96	21.5	35	15.0	**0.039**
	BPD	32	7.2	28	12.0	**0.036**
	BE/BO	24	5.4	10	4.3	0.529
	Chronic cough	12	2.7	11	4.7	0.169
	Other *	21	4.7	9	3.8	0.603
Comorbidities	Allergy ^+^	69	15.5	43	18.4	0.332
	AD	70	15.7	24	10.3	0.051
	Prematurity	54	12.1	41	17.5	0.053
	Developmental delay/neurological disorders ^+ +^	60	13.5	30	12.8	0.817
	Cardiovascular disorders ^+ ++^	12	2.7	6	2.6	0.922
	GI disorders ^+ +++^	21	4.7	12	5.1	0.809
Routine respiratory treatment	ICS	217	48.7	93	39.7	**0.027**
	BD	138	30.9	70	29.9	0.782
	Montelukast	46	10.3	18	7.7	0.266
	Preventive ABX	22	4.9	7	3	0.234
	CPT	44	9.9	20	8.5	0.576
	Antiallergic medications ^!^	26	5.8	14	6	0.936
Regular pulmonologist clinic visits		358	80.3	185	79.1	0.709
Prior hospitalization due to respiratory disorder		202	45.5	104	44.4	0.794

Abbreviations: BPD—bronchopulmonary dysplasia, BE—bronchiectasis, BO—bronchiolitis obliterans, AD—atopic dermatitis, GI—gastrointestinal, ICS—inhaled corticosteroids, BD—bronchodilators, CPT—chest physiotherapy, ABX—antibiotics. * Other—primary ciliary dyskinesis, neuroendocrine hyperplasia of infancy, pneumothorax, atelectasis. ^+^ Allergy—allergic rhinitis, allergic conjunctivitis, aeroallergens sensitivity, food allergies. ^+ +^ Neurological disorders—e.g., attention deficit disorder, trisomy. ^+ ++^ Cardiovascular disorders—e.g., congenital heart disease. ^+ +++^ GI disorders—e.g., celiac disease, inflammatory bowel disease, failure to thrive. ^!^ Anti-allergic medications—e.g., antihistamines, intranasal steroids. **Bold** indicates significant.

**Table 2:** Clinical status during lockdowns.

		Lockdown				
		First		Third		
		Patients (*n*)	Patients (%)	Patients (*n*)	Patients (%)	*p* Value
Respiratory symptoms	Decreased	133	33.0	58	27.4	0.063
	Unchanged	228	56.6	119	56.1	
	Increased	42	10.4	35	16.5	
Exacerbation frequency	Decreased	147	35.5	76	35.5	0.844
	Unchanged	232	56.0	117	54.7	
	Increased	35	8.5	21	9.8	
Use of reliever medications	Decreased	101	27.4	40	20.2	**0.006**
	Unchanged	222	60.3	115	58.1	
	Increased	45	12.2	43	21.7	
Medication adherence	Decreased	29	8.7	12	7.1	0.291
	Unchanged	238	71.3	113	66.9	
	Increased	67	20.1	44	26.0	

**Bold** indicates significant.

**Table 3:** Lockdown-related parameters.

		Lockdown Number				
		First		Third		
		Patients (*n*)	Patients (%)	Patients (*n*)	Patients (%)	*p* Value
Adherence to lockdown policies	Not at all	6	1.3	10	4.3	
	Satisfactory	109	24.4	86	37.1	**<0.001**
	Maximal	331	74.2	136	58.6	
Overall utilization of medical services	Decreased	57	16.7	26	13.8	0.598
	Unchanged	197	57.6	109	57.7	
	Increased	88	25.7	54	28.6	
Utilized medical services	Telephone	118	26.5	93	39.7	**<0.001**
	Email	31	7.0	70	29.9	**<0.001**
	Online	52	11.7	18	7.7	0.106
	ER	13	2.9	20	8.5	**0.001**
	Hospitalization	4	0.9	14	6.0	**<0.001**
	Decreased	7	1.6	5	2.3	
Screen time	Unchanged	83	19.1	46	20.7	0.736
	Increased	344	79.3	171	77.0	
	Decreased	236	53.9	120	53.8	
Physical activity	Unchanged	117	26.7	76	34.1	**0.025**
	Increased	85	19.4	27	12.1	
	Decreased	73	16.6	53	23.3	
Healthy eatinghabits	Unchanged	223	50.8	130	57.3	**0.001**
	Increased	143	32.6	44	19.4	
	Decreased	155	35.1	59	25.7	
Sleep duration	Unchanged	227	51.5	146	63.5	**0.011**
	Increased	59	13.4	25	10.9	

**Bold** indicates significant.

## Data Availability

The data presented in this study are available on request from the corresponding author. The data are not publicly available due to privacy of participants.

## Supplementary Materials

The following supporting information can be downloaded at: https://www.mdpi.com/article/10.3390/children9121862/s1, Supplementary File S1.
