# Supplementary material for: Lockdown Fatigue in Pediatric Respiratory Patients: Lessons from the First COVID-19 Year
Source: Children (Basel). 2022 Nov 30;9(12):1862. doi: 10.3390/children9121862 (PMC9776677; doi:10.3390/children9121862)
Supplement: Supplementary file 1 [file children-09-01862-s001.zip › children-2022098-supplementary.pdf]

Supplementary File S1

Anonymous questionnaire for caregivers regarding the clinical status of children with chronic respiratory disorders during mandatory national lockdown during the COVID-19 pandemic

First of all, we would like to thank you for your cooperation during the first lockdown, by answering our questionnaire, you have contributed to a better understanding of clinical status of children with underlying respiratory disorders as well as their caregivers` emotional status.
The following questionnaire is anonymous and concerns the long term clinical effects of lockdowns during the COVID-19 pandemic, on children with underlying respiratory disorders.
The questionnaire is short and will only require a few minutes of your time.
If you are using a mobile phone to complete the form, it is recommended that you place your phone in the horizontal position in order to visualize all the available answers.
By completing the form, you acknowledge that you had given your consent of to participate in our research.
Thank you in advance for your cooperation.

1. What is the date of birth of your child -dd/mm/year?
2. What is the sex of your child – Female /Male?
3. Number of individuals living in your home during lockdown period for COVID-19 pandemic?
4. To what extent were you able to follow lockdown restrictions during the lockdown period during COVID-19 pandemic?

a. Not at all
b. Minimally
c. Satisfactorily
d. Entirely

5. What is your child's respiratory disorder? (more than one answer can be marked)

a. Asthma/pre-school asthma/recurrent wheezing/spastic bronchitis
b. Pneumonia/recurrent pneumonia
c. Bronchopulmonary dysplasia

d. Bronchiectasis/bronchiolitis obliterans
e. Chronic cough
f. My child does not have a respiratory disorder
g. Other disorder (specify)

6. Does your child have any other chronic disorders?
a. No
b. Yes

7. If your child has additional chronic disorders, please specify (more than one answer can be marked)
a. Allergies (food/airborne allergens)
b. Atopic dermatitis
c. Cardiovascular diseases
d. Complications of prematurity
e. Developmental delay/other neurological disorders
f. Gastrointestinal disorders
g. Other disorder (specify)

8. Does your child receive any routine medical treatment for his/her respiratory disorder?
a. No
b. Yes

9. If routine treatment is provided, please specify (more than one answer can be marked)
a. Inhaled corticosteroids (Budicort, Flixotide, QVAR, etc.)
b. Inhaled long- or short-acting bronchodilators (Ventolin, Terbuline, Aerovent, Symbicort, etc.)
c. Montelukast/Singulair
d. Prophylactic antibiotics
e. Airway clearance techniques – chest physiotherapy/insufflation exsufflation cough assist
f. Antihistamine/anti-allergy treatment (Aerius, Telfast, Avamis, Flixonase, Steronase, etc.)

g. Other treatment (specify)

10. Was your child followed regularly by a pulmonologist in the last year?

a. No

b. Yes

11. How frequent were the pulmonologist follow-up visits?

a. Once a year/two years

b. 1-2 visits/ year

c. 3-4 visits/ year

d. 5-6 visits/ year

12. Was your child ever hospitalized due to his/her respiratory disorder?

a. No

b. Yes

13. If you marked "yes", please provide the number of hospitalizations of your child prior to COVID-19 pandemic.

14. Was your child infected with COVID-19 during the last year ?

a. No

b. Yes

15. In comparison to the state of your child during routine period, please share your opinion on changes that may have occurred during the lockdown period for COVID-19 pandemic:

	Significantly more	More	None	Fewer	Significantly fewer	Irrelevant
My child had respiratory symptoms (cough, running nose,						

difficulty breathing, wheezing)						
My child’s respiratory disorder was stable (exacerbation frequency						
I had used reliever medications, such as bronchodilators or inhaled corticosteroids						
I adhered to routine treatment						
I was anxious about my child’s respiratory disorder						
I felt that my child is at an elevated risk due to his/her respiratory disorder						
I consulted a medical professional regarding my child’s respiratory disorder						

16. If you consulted a medical professional during the lockdown period for the COVID-19 pandemic, how did you contact him/her? (more than one answer can be marked)

a. Telephone
b. Email
c. Online healthcare links
d. Regular visit at the clinic
e. Emergency room visit
f. My child was admitted to the hospital
g. I did not seek medical consultation/help

17. In comparison to normal times, please share your opinion on changes in the following lifestyle elements that may have occurred during the lockdown period for COVID-19 pandemic:

	Significantly more	More	None	Fewer	Significantly fewer	Irrelevant
My child`s leisure screen time						
My child`s physical activity						
My child's following a healthy diet						
My child`s sleep duration						
